# Development of a simple and high-yielding fed-batch process for the production of porcine circovirus type 2 virus-like particle subunit vaccine

**DOI:** 10.1186/s13568-019-0880-8

**Published:** 2019-10-11

**Authors:** Wenlong Cao, Hui Cao, Xiaoping Yi, Yingping Zhuang

**Affiliations:** 1 School of Biotechnology, East China University of Science and Technology, Shanghai, 200237 China; 2 Zhejiang EBVAC Biotech Co., Ltd., Hangzhou, 310018 Zhejiang China

**Keywords:** Virus-like particles, Porcine circovirus, Subunit vaccine, Fed-batch process, Baculoviral expression system, Feeding “cocktail”

## Abstract

The cap protein is encoded by the orf2 gene of porcine circovirus type 2 (PCV2) has the main antigen epitope of PCV2 and can form virus-like particles (VLPs), which are expressed in insect cells. PCV2-VLPs can effectively inhibit PCV2 replication as a subunit vaccine. In this study, a robust and reliable fed-batch process was successfully developed for the production of PCV2-VLPs by Sf9 cells. The feeding solution, feeding strategy, and cell density at infection were optimized to maximize the final PCV2-VLPs production yields. The cell density at infection and the volumetric PCV2-VLPs production reached 12 × 10^6^ cells/mL and 110 mg/L, respectively, which yielded 3- and 3.6-fold enhancements compared to the batch culture. The PCV2-VLPs produced in fed-batch culture were not different from the PCV2-VLPs produced in a batch culture in an immunity test. A highly efficient production process was produced for PCV2-VLPs subunit vaccines, which could provide an effective means for the industrial production of PCV2 vaccines.

## Introduction

Porcine circovirus type 2 (PCV2) is a malignant infectious pathogen for swine, potentially leading to PMWS (post-weaning pigs multisystemic wasting syndrome) (Gresham et al. 2000), PDNS (porcine dermatitis and nephropathy syndrome) (Cruz et al. 2018; Drolet et al. 1999), PNP (proliferative and necrotizing pneumonia) (Drolet et al. 2003), CT (congenital tremors) (Stevenson et al. 2001), PRDC (porcine respiratory disease complex) (Kim et al. 2003) and reproductive disturbances (Madson et al. 2008, 2009). The aforementioned conditions are collectively referred to as porcine circovirus diseases (PCVD) (Segalés et al. 2005). At present, this virus has caused significant losses in the pig industry.

The control of PCVD is based on management strategies, control of co-infections, and vaccination. The PCV2 vaccines have been reported to include inactivated vaccines (Pogranichniy et al. 2004), attenuated vaccines (Fenaux et al. 2004), a DNA vaccine (Kamstrup et al. 2004) and subunit vaccines (Kixmöller et al. 2008; Young et al. 2008). Due to the complexity of virus prolification in vitro and relatively low viral titers, the production of traditional inactivated vaccines and attenuated vaccines has been limited. Therefore, the development of subunit vaccines has become an important alternative direction.

The PCV2 genome contains two major ORFs flanking the origin of replication: orf1 and orf2 (Franzo et al. 2016). Orf1 encodes two viral replication-associated proteins, Rep and Rep’ by differential splicing (Cheung 2006, 2007; Grau-Roma et al. 2008). The Orf2 encoding cap protein is a viral structural protein, which has the ability to bind to the host cell receptors (Khayat et al. 2011; Lekcharoensuk et al. 2004; Misinzo et al. 2006). The cap protein is the primary immunogenic protein of PCV2 (Blanchard et al. 2003; Mahé et al. 2000) and thus has been the target for development of vaccines and serodiagnostic assays for tracking PCV2-specific immune responses. Fort and colleagues (Fort et al. 2008) evaluated a subunit vaccine containing the PCV2 cap protein. PCV2 was expressed in the baculovirus by using a challenge model with four different PCV2 proteins from different genotypes and geographic origins (Opriessnig et al. 2017). The vaccine prevented the development of viremia in all cases, as well as significantly decreased nasal and faecal shedding of the virus. Additionally, the vaccine effectively stimulated the expression of PCV2-specific neutralizing antibodies, even in the presence of maternal antibodies.

The baculoviral expression system (BEVS) is a promising platform for mass production of vaccines. BEVS has recently been used in the manufacture of both veterinary and human vaccines extensively, and more than 10 BEVS-derived products are now either commercially available or in clinical trials. BEVS has the advantages of high efficiency and easy amplification (Martinez-Solis et al. 2019). The PCV2 cap protein manufactured by BEVS can assemble to a virus like particle spontaneously, which stimulates humoral immunity, secretes neutralizing antibodies, and also stimulate cellular immunity. The product has a very good virus immune effect.

For the large-scale production of pharmaceutical proteins based on the BEVS, many reports indicated that the unit cell yield could be significantly decreased when the cell density of infection (CDI) exceeds a certain threshold, which is called “the density effect”. Although this phenomenon has been extensively studied, its mechanism is still not fully understood. The density effect is usually considered a result of nutrient depletion during the viral infection, and it may also be accumulation of by-products. Bernal et al. (2009) investigated the cell metabolism of insect cells and observed that the accumulation of the by-products and the nutrient consumption could not explain the phenomenon of decline in the yield-inoculated virus under high cell density. The density effect was likely to be the result of cell metabolism. When the cell density increased, the amino acids in the TCA cycle and glycolysis metabolism decreased and central metabolism gradually weakened. Previous studies have also indicated that the yield of recombinant protein was reduced by the occurrence of nutritional depletion. Even so, the “density effect” can be reduced or offset through partial medium change or adding some specific nutrition (Carinhas et al. 2010).

Fed-batch cultivation is an intermittent supplementary nutrient to prevent nutrient depletion. Fed-batch cultivation is more convenient and economical than perfusion culture, and it is more operable. BEVS is a transient expression system, after inoculation with the virus, the cells can be cleaved and the time of producing a protein is very short. Moreover, the insect cells are not sensitive to the accumulation of by-products, osmotic pressure and pH, so the scale production of BEVS is more suitable for fed-batch cultivation. Many reports have confirmed that fed-batch cultivation can significantly increase the cell density and/or production of the product (Fu et al. 2018). There was no significant difference in the yield of the final product in the fed-batch cultivation of the onetime supplementary yeast hydrolysate and amino acid mixture compared with the batch culture. The highest cell density can be reached by 30 × 10^6^ cells/mL by adding more abundant nutrition, including glucose, tyrosine, vitamins and trace elements. However, due to the presence of the density effect, the infection of the high cell density was not successful. The highest cell density of infection was 12 × 10^6^ cells/mL, which can maintain a high production yield.

In the present study, serum-free medium of Sf9 cells’ feeding medium was optimized by the DOE (Design of Experiment) method to achieve high cell density. In the fed-batch culture, we could achieve a high cell density of infection by optimizing the feeding strategy, and effectively alleviating the density effect. This study provides an efficient and easy method for the production of PCV2-VLPs in the bioreactor.

## Materials and methods

### Cell maintenance and viral stocks preparation

Cells were routinely grown and maintained in 250 mL (50 mL working volume) shake flasks (Corning Inc, Suzhou, China) a in serum-free medium (SFM) at 28 °C and 110 rpm. The recombinant baculoviruses were amplified by infecting Sf9 cells and harvesting the cells for 4 days post-infection, as previously described by Nawagitgul et al. (2000). The virus stock solution titres were determined by a plaque assay, as previously described by O’Reilly et al. (LaBarre and Lowy 2001). The trypan blue exclusion test is used to determine the number of viable cells present in a cell suspension and the cell density and viability was visually examined in haemocytometer (Strober 2001). Cell concentration was determined by haemocytometer cell counts (Anxin Optical, Shanghai, China) and cell viability was evaluated by trypan blue dye exclusion (Sigma Aldrich, CA, USA) in 0.4% PBS.

### Molecular cloning and expression of viral genes

The baculovirus expression system (Life technologies, NY, USA) was used to express the *orf2* gene. Viral DNA was isolated from PK-15 cells (ATCC CCL31) infected with the SD-1 strain of PCV2 by a QIAamp DNA Mini Kit (Qiagen, Germany). Next, the *orf2* gene was amplified cloned with following primers P1:5′-TATGGATCCACGTATCCAAGGAGGCGTTACCGGAGAAG-3′, P2:5′-CGGCTGCAGTTAGGGTTTAAGTGGGGGGTCTTTAAGAT-3′), which were designed based on the sequence of the PCV2 in GenBank (No. HM776451) and *Bam*HIat 5′ end of P1 and *Pst*Isites (underlined) at P2.

PCR-cloned *orf2* was ligated into pFastBac 1 and double digested with *Bam*HIand *Pst*I. The resulting plasmid was then introduced into *E. coli* DH5α competent cells. PCR screening for positive transformed colonies were conducted and plasmids extracted from those positive colonies were digested with *Bam*HI/PstI. The plasmids were then sequenced (Invitrogen, Shanghai, China) to determine the orientation and integrity of the inserted sequences. Recombinant baculovirus carrying the *orf2* gene were constructed according to the manufacturer’s instructions (Bac-to-Bac baculovirus expression system). Briefly, *E. coli* DH10Bac containing the baculovirus shuttle vector (bacmid) and helper vector was transformed with the recombinant plasmid pF-*orf2*. Within *E. coli* DH10Bac, the *orf2* gene was transposed into the bacmid. The colonies of *E. coli* containing recombinant bacmid were collected by blue/white selection. The recombinant bacmid DNA was isolated, purified and transfected into Sf9 cells to yield AcMNPV carrying the PCV2 *orf2* gene, referred to as Ac-*orf2*, under the control of the polyhedrin promoter. Primary stock of Ac-*orf2* was harvested at 72 h post-transfection. Expression of the *orf2* gene of PCV2 was confirmed by a western blot using a swine anti-PCV2 serum (Boshide Corporation, Wuhan, China) and HRP-labeled goat anti-swine IgG (KPL Corporation, USA).

### Screening of nutrients using the Plackett–Burman design

The Plackett–Burman design for 7 variables, which includes 7 nutrients at two levels (+ 1 and − 1) (Additional file 1: Tables S1, S2) (Plackett and Burman 1946) was used for screening feed medium components. They were tested for the significance of promoting cell growth. The nutrients were hydrolysate (yeast extract and rice extract), amino acids, vitamins, inorganic salts, lipids, disaccharides (maltose and sucrose), and organic acids (pyruvate and α-ketoglutarate). In the experimental design, each row represents an experiment and each column represents an independent variable (Additional file 1: Table S2). The signs “+” and “−” represent the two different levels (higher and lower) of the independent variable under investigation. In the feed medium, glucose and glutamine are the two base nutrients for primary metabolism. Cell growth is significantly impacted when these nutrients are limited. Therefore, glucose and glutamine were fixed in feed medium and their concentrations were determined by consumption (the concentration are 5 g/L and 8 mM respectively) before feeding.

Sf9 cells were resuspended at a density of 4 × 10^6^ cells/mL as in the mother flask. From these suspensions, triplicate 10 mL cultures in 50 mL shake-flasks were prepared as one group; a total of 12 groups were prepared. The feed supplements were added to each group culture according Additional file 1: Table S1 and sampled daily for the cell density.

### The effects of cell density at infection for PCV2-VLPs production in batch culture in a 1.5 L bioreactor

The batch culture was performed in three controlled 1.5 L bioreactors (Guoqiang Inc, Shanghai, China) with a 600 mL working volume. The batch bioreactor cultures were started by inoculating the cells at a viable cell density (VCD) of 0.7 × 10^6^ cells/mL. The cell density of infection was 2 × 10^6^ cells/mL, 4 × 10^6^ cells/mL and 6 × 10^6^ cells/mL in three bioreactors using an MOI of 1.

### Determination of the CDI in fed-batch cultures in a 1.5 L bioreactor

The PCV2-VLPs were expressed in three controlled 1.5 L bioreactors (Guoqiang Inc, Shanghai, CHN) with a 600 mL working volume as above. The cocktail solution was introduced into the bioreactors at a CDI of 4 × 10^6^ cells/mL (mid-exponential growth phase). They were infected at a target cell density of infection of 4.0 × 10^6^ cells/mL, 8.0 × 10^6^ cells/mL, 10 × 10^6^ cells/mL, 12 × 10^6^ cells/mL, and 14 × 10^6^ cells/mL with recombinant baculovirus using a MOI of 1. A batch culture of infected sf9 cells at a VCD of 4 × 10^6^ cells/mL was run in parallel to the fed-batch experiments as a control.

### Western blot

The cellular proteins from cell suspension were separated by sodium dodecyl sulfate-polyacrylamide gel electrophoresis (SDS-PAGE, 12% acrylamide). After electrophoresis, proteins were transferred onto PVDF membranes (Millipore). The PVDF membrane was soaked in 5% milk before incubation with swine anti-PCV2 serum (1:100) overnight at 4 °C. Detection was achieved with a peroxidase-conjugated goat anti-swine Ab (KPL), followed by the addition of an ECL substrate (Amersham) and Chemiluminescence imaging was taken by ChemiDoc Touch (Bio-Rad, USA).

### Analysis the concentration of cap protein by ELISA

Infected cultures were sampled (4 mL) daily and stored at − 80 °C. The recombinant cap protein contents in all preparations were determined by ELISAs. Briefly, the cell suspension was disrupted by ultrasonication on ice at 200 W of power with 3-second bursts and 3-second intervals between bursts. The lysate was obtained by centrifugation at 12,000×*g* at 4 °C for 15 min. The suspension was harvested and further diluted 300-fold in carbonate buffer solution (pH = 9.6). 100 μL of suspension was added per well to a Corning^®^ 96 Well White Polystyrene High Bind Stripwell™ Microplate (Corning Inc, MA, USA) and incubated at 4 °C overnight. Prior to addition of the antibody layers, plates were blocked for 1 h at room temperature with 1% collagen in PBS. The reagents were used at 100 mL/well and incubations, unless otherwise stated, were for 1 h at 37 °C. The washes were performed with ELISA buffer (0.5 M NaCl, 15 mM Na_2_HPO_4_, 2 mM KH_2_PO_4_, and 0.05% Tween 20). The plates were first incubated with PCV2 cap protein monoclonal antibody diluted (1:2000) in PBS with 0.05% Tween 20. The secondary antibody, horseradish peroxidase (HRP)-conjugated sheep anti-mouse IgG (KPL, USA), was diluted at 1:3000 in PBS–0.05% Tween 20. The ELISA plates were developed with Ultra TMB-ELISA substrate (Thermo Scientific, MA, USA) for 5 min at room temperature. The reaction was stopped with 1 M sulfuric acid, and the absorbance at 450 nm was determined with a microplate reader. The Sf9 cells infected by wild baculovirus was the negative control and the standard curve was plotted by the commercial PCV2 cap protein standard substance.

### Immunization of mouse

Sf9 cells were infected by the Ac-*orf2* and the cultures were harvested at 72 h post-infection (hpi). The cultures were disrupted by ultrasonication and the cap protein concentration was determined with an ELISA. The PCV2-VLPs subunit vaccine was manufactured by the following method: 10 μg of cap protein was emulsified with montanide ISA 28 VG adjuvant (Seppic, France) at a volume ratio of 3:1.

A total of 40 healthy special pathogen free (SPF) mice aged 6–8 weeks were randomly allotted into four groups (10 mice per group). Mice were immunized in enterocoelia. Group 1 was immunized with the commercial PCV2 inactived vaccine (Lot: 1401001, Qilu Animal Healthcare, China) as a positive control. Group 2 was immunized with the PCV2-VLPs subunit vaccine. Group 3 was immunized with Sf9 cells, which were infected by wild baculovirus, as a negative control. Group 4 was immunized with PBS as a blank control. Twenty-one days after vaccination, all mice were inoculated with 0.45 mL of the PCV2-ZJ strain (107 TCID50/mL; obtained from the National Institute of Veterinary Drug Control, PR China; sequence number in GenBank: AY686764.1). The mice were subsequently euthanized 21 days later and the spleen was harvested to detect the PCV2 virus. Briefly, 0.05 g spleen tissue was added to 1 mL of MEM medium, homogenized by grinding, and went through 3 freeze/thaw cycles at − 80 °C. Next, the samples were centrifuged for 10 min at 12,000 rpm and the spleen tissue supernatant was filtered. The PK15 cells were seeded in a 12-well microplate with 3 × 105 cells per well and 0.15 mL spleen tissue supernatant was added. The PK15 cells were cultured for 48–60 h and the quantity of PCV2 virus was determined by IFA as described by Truong et al. (2001). The vaccines were used only if the PCV2 virus was detectable in the spleen tissue of at least 7 mice of the blank and negative control groups. The vaccines were not used if the PCV2 virus was not detectable in at least 7 mice of the vaccine-treated group.

### Statistical analysis of the data

The use of statistically planned experimentation is to identify the significant variables and their corresponding coefficients, so that the levels of variables can be managed to obtain a desired output. Hence, the coefficients, sum of squares in percentage (SS%) and confidence interval (CI) (Thomas et al. 1987) were analysed using the experimental results of the maximum cell density. Using the software package Minitab 16 (Minitab Inc, USA) the experimental plan, the analysis and the results were obtained.

## Results

### Confirmation of the expression of cap protein and the formation of PCV2-VLPs

**Fig. 1:**
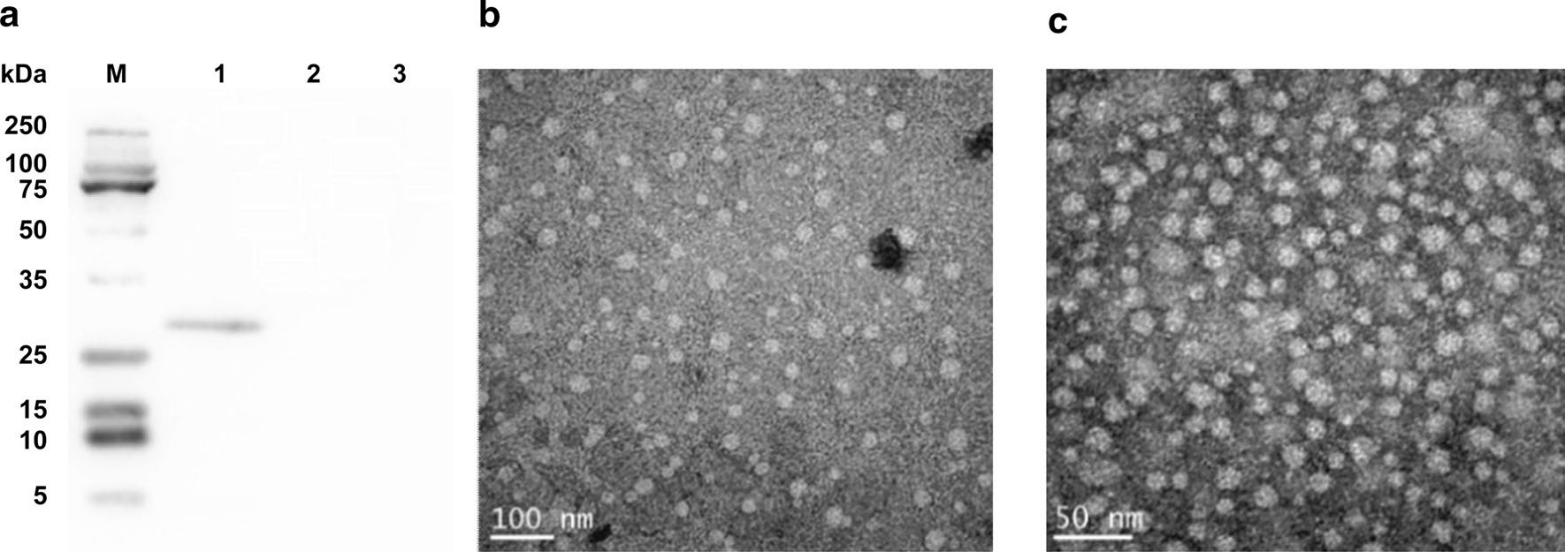
**a** Immunoblot of recombinant cap proteins showing that PCV2 cap protein was correctly expressed Sf9 cells infected with recombinant Ac-orf2 are shown in lane 1, wildtype AcMNPV-infected Sf9 cells are in lane 2, and uninfected Sf9 cells are in lane 3. Lane M contained standard molecular mass markers. Transmission electron micrographs of partially purified PCV2-VLPs expressed in insect cells (**b**) and purified PCV2 particles (**c**)

A single protein with the molecular weight of about 27 kDa was detected in the Ac-orf2-infected Sf9 cells, but not in the wildtype AcMNPV-infected and uninfected Sf9 cells (Fig. 1a), which suggests that the PCV2 orf2 gene was expressed in Sf9 cells. When the cap protein in insect cells was partially purified, numerous virus-like particles were detected by negative staining electron microscopy (Fig. 1b, c). The self-assembled particles were of similar morphology to the PCV2 virions. Both PCV2 particles and self-assembled particles were approximately 20 nm in diameter. The cap protein in the PCV2-VLPs was purified by sucrose gradient centrifugation and the total cap proteins in the cell lysis suspension before the purification was detected by the ELISA method. The results indicated that more than 95% of the cap proteins in the cell lysis suspension could assemble into VLPs. The expression level of PCV2-VLPs was indirectly quantified using the concentration of cap proteins.

### Screening the components of feeding solutions

The average cell density of each group in the Plackett–Burman experiment are shown in Additional file 1: Figure S1 and the statistical analyses are shown in Additional file 1: Table S3. Three (hydrolysate compounds, disaccharide and organic acid compounds) out of 7 variables formed an 84.1% sum of squares (SS%) implying that these variables influenced the cell growth and maximum cell density. Disaccharide and organic acid compounds had negative coefficients while hydrolysate compounds showed positive coefficients. Additionally, the base medium had high concentrations of those four components, which were enough to support Sf9 cells growth to 1.5 × 10^7^ cells/mL.

### Optimizing the feeding medium components using the single factor design

**Fig. 2:**
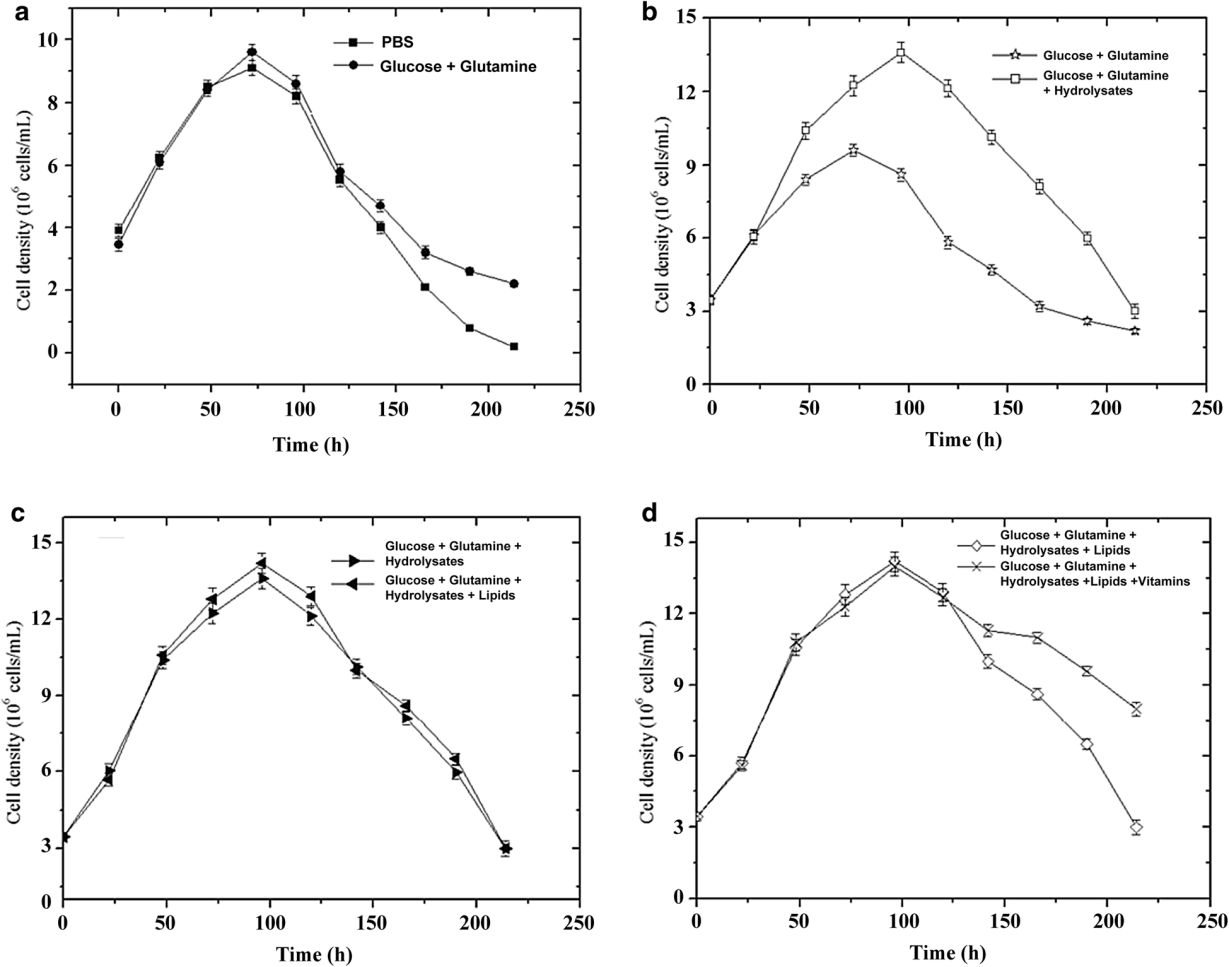
Effects of glucose, glutamine, hydrolysated, lipids and vitamins on the cell growth viability maintenance. **a** Compared the cells fed with PBS and glucose+glutamine only, **b** compared the cells fed with glucose+glutamine and glucose+glutamine + hydrolysates, **c** compared with the cells fed with glucose + glutamine + hydrolysates and glucose+glutamine + hydrolysates+lipids, **d** compared with the cells fed with glucose+glutamine + hydrolysates+lipids and glucose + glutamine + hydrolysates + lipids + vitamins

In order to confirm the results of Plackett–Burman design and optimize the feeding component, hydrolysates, inorganic salts, vitamins, lipids were examined individually to determine their contribution to cell growth and viability maintenance. The feeding was added at a cell density of 4 × 10^6^ cells/mL and each component are the same components shown in Additional file 1: Table S1. The value in each single-factor trial was an average of triplicates. Based on the Plackett–Burman design results, the feeding medium was composed of glucose, glutamine, hydrolysates, lipids and vitamins (Table 1). A comparison of feeding with PBS and feeding with glucose and glutamine illustrate that the maximum cell density did not increased when the cells were fed with glucose and glutamine only (Fig. 2a). As shown in Fig. 2b, hydrolysates promote cell growth as the maximum cell density reached 12.9 × 10^6^ cells/mL in group of feeding with glucose, glutamine and hydrolysates, which was about 1.4 times as high as feeding with glucose and glutamine only. As shown in Fig. 2c, it was illustrated that the addition of lipids had little effects on cell growth and viability maintenance (Fig. 2c). When feeding with the combination of glucose, glutamine, hydrolysates and lipids, the cells have a better shape: the cells are round and the edge is smooth, while the edge of the cells feeding with glucose, glutamine, hydrolysates only were rougher (Additional file 1: Figure S2). It is maybe that the synthesis of the cell membrane was disrupted in the cells. Indeed, insect cells have incomplete lipid metabolism (Agathos 2007) and need lipid supplementation. The lipids in the initial medium may not be sufficient for high cell densities. As shown in Fig. 2d, it was illustrate that the addition of a high concentration of vitamins affected cell viability maintenance.

**Table 1:** Components of the feed medium

Variables	Composition of the solutions used in the cocktail
Glucose	5 g/L glucose
Glutamine	8 mM glutamine
Hydrolysate	2 g/L yeast extract
Lipids^a^	Cholesterol, tocopherol acetate, cold liver oil fatty acid methyl esters, Tween 80 and Pluronic F-68
Vitamins^b^	Thiamine·HCl, riboflavin, d-calcium pantothenate
	Pyridoxine·HCl, para-aminoben, nicotinic acid,
	Zoic acid, i-inositol, biotin, choline chloride,
	Vitamin B12, folic acid

^a^Lipids mixture (Sigma-Aldrich, cat. L5146)

^b^Vanderzant vitamin mixture for insects (Sigma-Aldrich, cat.V1007)

### Effects of the fed-batch culture on cell growth and PCV2-VLPs production

**Fig. 3:**
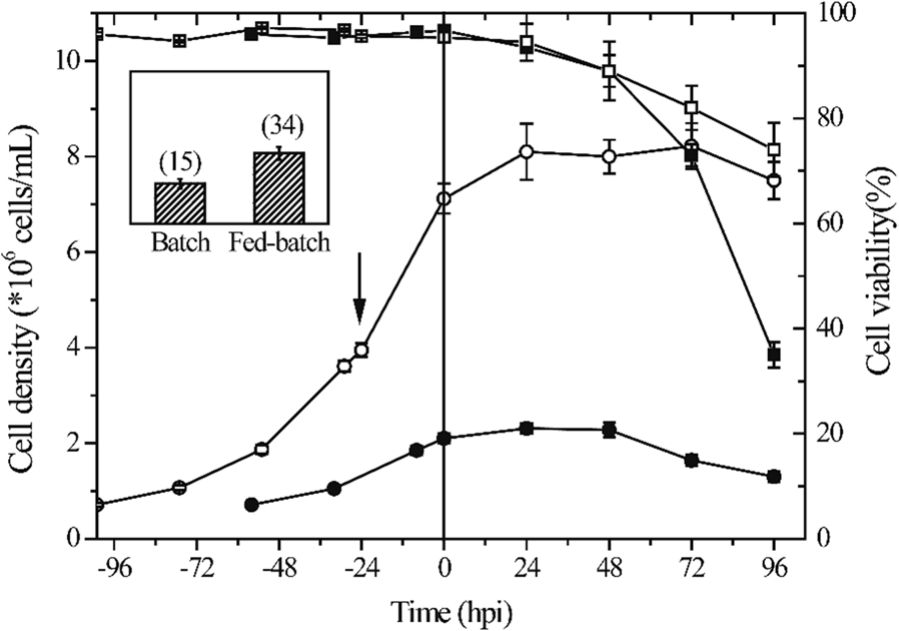
Cell growth profile and PCV2-VLPs production of the infected Sf9 cells from fed-batch and control batch cultures in shake-flask experiments. For the fed-batch cultures, sf9 cells were infected by the baculovirus Ac-*orf2*, at approximately 7 × 10^6^ cells/mL at an MOI of 1. The feed was added when the cell density was 4.0 × 10^6^ cells/mL and at the time of infection, as shown by the solid vertical arrows. Closed and open symbols represent the batch and fed-batch cultures, respectively; total cells (circle), cell viability (squares). The insert shows the volumetric PCV2-VLPs production expressed in mg/L for batch and fed-batch cultures after 96 hpi. Data represents the average of three independent experiments. Error bars represent standard deviation

Using the Ac-orf2 strain as the model, the shake-flask experiments were conducted and the varies feeding solution were evaluated in order to improve the cell density and PCV2-VLP production. When the cell density reached to 4.0 × 10^6^ cells/mL the feeding solution was added to the culture at a single pulse. A batch culture of infected Sf9 cells at a CDI of 2 × 10^6^ cells/mL was run in parallel to the fed-batch experiments as a control. The feed additions sustained a continue growth and infection at a CDI of 7.0 × 10^6^ cells/mL (Fig. 3). In the fed-batch cultures, the cell viability was still reached to 70% at 96 h post-infection (hpi), that is significantly higher than which obtained from the batch cultures. Enhancement of the cell density and viability at infection also resulted in increasing the PCV2-VLPs yields in the fed-batch cultures. PCV2-VLPs yields reached to 39 mg/L (see insert of Fig. 3), which is more than 2.5-fold increase over the batch control.

### Determination of the peak cell density for PCV2-VLPs production in 1.5 L batch culture

**Fig. 4:**
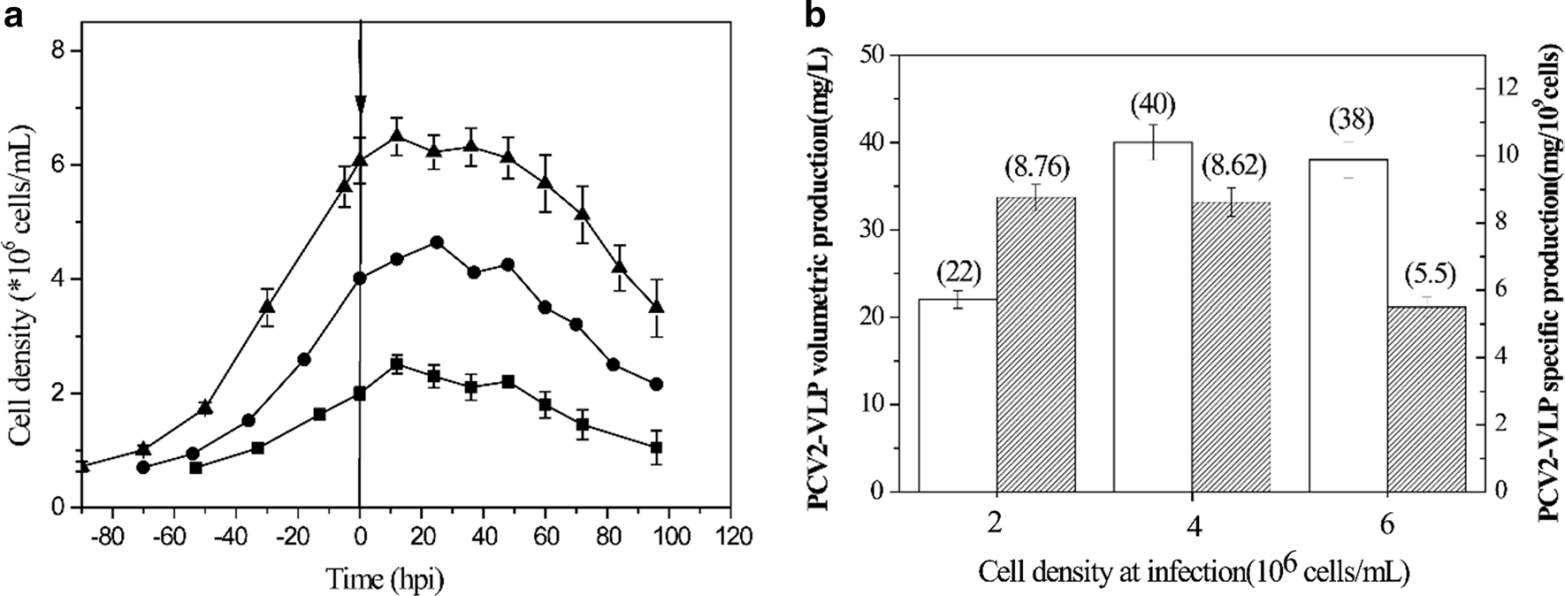
Production of PCV2-VLP in 1.5 L bioreactor batch cultures. The CDI ranged from 2 to 6.0 × 10^6^ cells/mL and the infection time is indicated by the vertical arrow. Data represents the average of three bioreactor runs. Error bars represent standard deviation. **a** Cell growth profile. **b** PCV2-VLP volumetric production (white bars) and specific production (shaded bars)

**Fig. 5:**
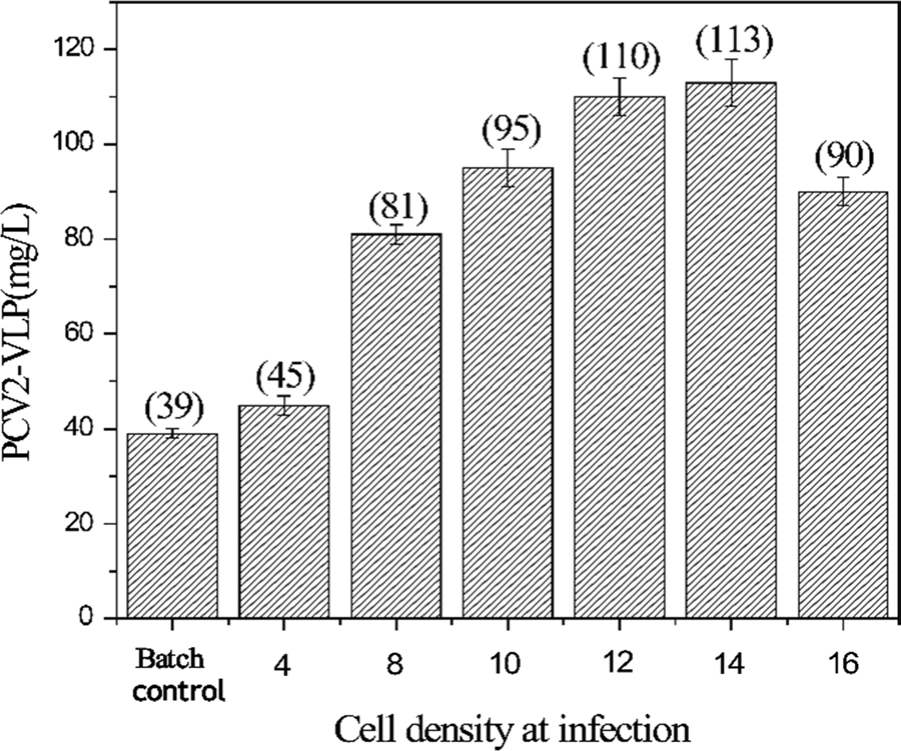
Effects of cell density at infection (CDI) on PCV2-VLPs production in 1.5 L bioreactor fed-batch cultures. One feed was added to the cells at a cell density of 4.0 × 10^6^ cells/mL. Sf9 cells were infected at different cell density ranging from 4.0 × 10^6^ to 16 × 10^6^ cells/mL. The batch control cells were infected at a CDI of 4 × 10^6^ cells/mL. Values are an average of three independent experiments. Error bars represent standard deviation

The batch culture was to induce PCV2-VLPs protein production in the 1.5 L bioreactor. Several CDIs ranging from 2 to 6.0 × 10^6^cells/mL were investigated. The cell growth curve and the PCV2-VLPs yield is shown in Figs. 4 and 5. The specific protein yields did not decrease when the CDI increased from 2 × 10^6^ to 4 × 10^6^ cells/mL and the volumetric PCV2-VLPs yields increased from 22 to 40 mg/L. When the CDI was increased to 6 × 10^6^ cells/mL, the volumetric PCV2-VLPs yields did not increase due to the specific protein yields that dropped 36%. The volumetric yield (VY, g/mL) initially increased linearly with CDI from 2 × 10^6^ to 4 × 10^6^ cells/mL, then reached a maximum, and declined rapidly at a CDI of 6 × 10^6^ cells/mL, when the “density effect” arose. The best CDI for the batch culture was 4 × 10^6^ cells/mL and which made this infection strategy the control for subsequent experiments.

### Determination of the peak CDI for PCV2-VLPs production in the fed-batch culture

The feeding solution that was developed was used in the rest of the study to further enhance the fed-batch process by optimizing the cell density at infection. Several CDIs ranging from 4 × 10^6^ to 16.0 × 10^6^ cells/mL were investigated. The feeds were added to the culture at a cell density of 4 × 10^6^ cells/mL to avoid potential nutrient limitations. A similar cell density used in batch production. The result is shown in Fig. 5. Within a certain range, the production of PCV2-VLPs increased linearly with CDI: when CDI increased to 12 × 10^6^ cells/mL, the volumetric yield of PCV2-VLPs increased by 2.82 times, when compared with the control batch culture, which reached 110 mg/L. When the CDI reached 14 × 10^6^ cells/mL, the volumetric yield of protein only slightly improved and the yield had a significant decline when the CDI was 16 × 10^6^ cells/mL and only reached 90 mg/L. Taking into account the process of stability and ease of operation, the optimal CDI was determined at a density of 12 × 10^6^ cells/mL.

### Verification the protection of PCV2-VLPs produced in fed-batch culture

Recombinant PCV2-VLPs produced in the fed-batch culture and batch culture in a 1.5 L bioreactor were harvested for the preparation of PCV2 subunit vaccines. The potency for the protection of PCV2 infection was tested in mice. The result is summarized in Table 2.

**Table 2:** The protection ratio of the subunit vaccine prepared by the PCV2-VLPs produced in different processes for the PCV2 virus infection in mice

Production process	Lot	Vaccines	Protection ratio (negative/all)
Batch culture	1	5.5 μg PCV2-VLPs	10/10
	2	5.5 μg PCV2-VLPs	10/10
	3	5.5 μg PCV2-VLPs	9/10
Fed-batch culture	1	5.5 μg PCV2-VLPs	9/10
	2	5.5 μg PCV2-VLPs	9/10
	3	5.5 μg PCV2-VLPs	10/10
Positive control	1	Commercial inactived vaccine	9/10
Negative control	1	Sf9 cells infected by wild baculovirus	2/10
Blank control	1	PBS	2/10

As shown in Table 2, the PCV2-VLPs quality, as determined by the immune protection test, was sufficient for all three lots, in batch culture or fed-batch cultures. This demonstrates that no significant differences were observed in the protection effect of the PCV2-VLPs produced in the fed-batch culture with the high cell density infection strategy or conventional production process. The fed-batch culture process was successful and could be used for the large-scale production of PCV2-VLPs subunit vaccines.

A feeding cocktail containing 5 types of compounds was developed using the compounds listed in Additional file 1: Table S2. This complex feed was first used to demonstrate growth up to 21 × 10^6^ cells/mL of Sf-9 insect cells (data not shown). We verified the effect of the feed medium on the expression of PCV2-VLPs in the flask. The cocktail feed was added when the cell density was 4 × 10^6^ cells/mL. A sustained production at a cell density of 7.0 × 10^6^ cells/mL, represents more than a 3.5-fold increase over the batch cultures. The PCV2-VLPs yield increased 2.6-fold, indicating that the serum-free feed media was effective for PCV2-VLPs production.

Any efficient fed-batch strategy relies on the appropriate design of the feed medium and the feeding regimen. Taking into account the operability of an industrial scale process, we simplified the feeding regimen. We used one single pulse feed at the fixed cell density of 4 × 10^6^ cells/mL because the cells were the most robust at this point and could sustain a high cell growth rate, even if the culture environment changed intensely with feeding operation. The CDI was optimized in the 1.5 L bioreactor. The optimized CDI was 12 × 10^6^ cells/mL. When compared to the control, the optimized CDI increased 3 times and the specific volume of protein yield increased 2.8 times, reaching 110 mg/L.

Three batches of fed-batch culture were implemented on 1.5 L bioreactor successfully and the recombinant PCV2-VLPs produced in the batch culture and fed-batch culture were harvested. The PCV2 subunit vaccines, which were made by the harvested PCV2-VLPs and produce in two different process, displayed similar immune protective effects (Table 2). This demonstrates that the quantity of the PCV2-VLPs produced in fed-batch culture has not been changed. It suggests that the fed-batch culture process developed in the present study was stable, robust and reliable.

## Discussion

In the present study, an efficient and robust method for the production of PCV2-VLPs in the bioreactor was performed. Serum-free medium of Sf9 cells’ feeding medium was optimized to achieve high cell density. And we could also achieve a high cell density of infection by optimizing the feeding strategy in the fed-batch culture.

During the screening the components of feeding solutions, the addition of organic acids may lead to increased osmotic pressure; however, the reason sf9 cell growth is suppressed by disaccharides in not clear. The effects of the other four variables, animal acid compounds, vitamin compounds, lipids were found to not be significant, but this does not rule out their potential role in cell growth.

The large-scale production of PCV2-VLPs has not been reported, especially those from insect cells that are infected at high cell density (Duan et al. 2019; Wu et al. 2016). It has been well established that mitigating metabolic limitations during the infection-production phase is more challenging than that for cell growth. This phenomenon referred to as the “cell density effect”, which has been extensively documented by others for insect and human cell production systems infected by viral vectors (Chaves et al. 2018; Mlera and Bloom 2019). The specific virus production may drop sharply when infected at a high cell density. In our experiment, the volumetric yield (VY, g/mL) of PCV2-VLPs initially increased linearly with CDI, then reached a maximum, and finally declined rapidly at higher CDIs. The bioprocess optimization for VLPs production using the BEVS in a bioreactor was rarely reported in previous studies. The PCV2-VLPs productivity is usually very low when using the conventional bioprocess biotechnology. To avoid the “cell density effect”, the CDI is very low. In order to establish an efficient and economic PCV2-VLPs vaccine production process. Our strategy was used to improve PCV2-VLPs yield by increasing the cell concentrations in fed-batch cultures while maintaining the cell specific production yield. By utilizing this strategy, we explored an efficient PCV2-VLP2 manufacture process which characterized the high cell density infection and high specific protein yield, which was implemented in a 1.5 L bioreacter for 3 batches of stable culture.

It was found that even though the feed was added and the cells were infected at a higher cell density (more than 7 × 10^6^ cells/mL) in the flask culture, the PCV2-VLPs yield was not increased. It may not have been the origin of nutrient limitation (the main nutrition such as glucose, glutamine, animo acids and others were sufficient, data not shown). This was probably due to an oxygen limitation. There is evidence that the consumption of oxygen increased significantly when Sf9 cells are infected with baculovirus (Weiss et al. 1982).

In conclusion, A feeding cocktail containing 5 types of compounds (glucose, glutamine, hydrolysates, lipids and vitamins) was developed using the compounds listed in Additional file 1: Table S2. This feeding medium was stable, reliable, and necessary to maintain high cell activities and PCV2-VLP output. The feeding strategy and cell density at infection were optimized to maximize the final PCV2-VLPs production yields. The cell density at infection and the volumetric PCV2-VLPs production reached 12 × 10^6^ cells/mL and 110 mg/L, respectively, which yielded 3- and 3.6-fold enhancements compared to the batch culture. The PCV2-VLPs produced in fed-batch culture were not different from the PCV2-VLPs produced in a batch culture in an immunity test. Thus, the PCV2-VLPs produced in a fed-batch culture and in a batch, culture have similar immune protective effects. The culture process was successfully carried out in 3 batches in a 1.5 L reactor. A highly efficient production process was produced for PCV2-VLPs subunit vaccines, which could provide an effective means for the industrial production of PCV2 vaccines.

## Supplementary information

**Additional file 1.** Additional tables and figures.

## Data Availability

All data analysed throughout this study are shown in this article. All strains and reagents were purchased in microbial collections or in companies, respectively, specified in the text.

## References

[CR1] Agathos SN (2007). Development of serum-free media for *lepidopteran* insect cell lines. Methods Mol Biol.

[CR2] Bernal V, Carinhas N, Yokomizo AY, Carrondo MJ, Alves PM (2009). Cell density effect in the baculovirus-insect cells system: a quantitative analysis of energetic metabolism. Biotechnol Bioeng.

[CR3] Blanchard P, Mahe D, Cariolet R, Keranflec’h A, Baudouard M, Cordioli P, Albina E, Jestin A (2003). Protection of swine against post-weaning multisystemic wasting syndrome (PMWS) by porcine circovirus type 2 (PCV2) proteins. Vaccine.

[CR4] Carinhas N, Bernal V, Monteiro F, Carrondo MJT, Oliveira R, Alves PM (2010). Improving baculovirus production at high cell density through manipulation of energy metabolism. Metab Eng.

[CR5] Chaves LCS, Ribeiro BM, Blissard GW (2018). Production of GP64-free virus-like particles from baculovirus-infected insect cells. J Gen Virol.

[CR6] Cheung AK (2006). Rolling-circle replication of an animal circovirus genome in a theta-replicating bacterial plasmid in *Escherichia coli*. J Virol.

[CR7] Cheung AK (2007). A stem–loop structure, sequence non-specific, at the origin of DNA replication of porcine circovirus is essential for termination but not for initiation of rolling-circle DNA replication. Virology.

[CR8] Cruz TF, Magro AJ, de Castro A, Pedraza-Ordonez FJ, Tsunemi MH, Perahia D, Araujo JP (2018). In vitro and in silico studies reveal capsid-mutant porcine circovirus 2b with novel cytopathogenic and structural characteristics. Virus Res.

[CR9] Drolet R, Thibault S, D’Allaire S, Thomson JR, Done SH (1999). Porcine dermatitis and nephropathy syndrome (PDNS): an overview of the disease. J Swine Health Prod.

[CR10] Drolet R, Larochelle R, Morin M, Delisle B, Magar R (2003). Detection rates of porcine reproductive and respiratory syndrome virus, porcine circovirus type 2, and swine influenza virus in porcine proliferative and necrotizing pneumonia. Vet Pathol.

[CR11] Duan J, Yang D, Chen L, Yu Y, Zhou J, Lu H (2019). Efficient production of porcine circovirus virus-like particles using the nonconventional yeast *Kluyveromyces marxianus*. Appl Microbiol Biotechnol.

[CR12] Fenaux M, Opriessnig T, Halbur P, Elvinger F, Meng X (2004). A chimeric porcine circovirus (PCV) with the immunogenic capsid gene of the pathogenic PCV type 2 (PCV2) cloned into the genomic backbone of the nonpathogenic PCV1 induces protective immunity against PCV2 infection in pigs. J Virol.

[CR13] Fort M, Sibila M, Allepuz A, Mateu E, Roerink F, Segalés J (2008). Porcine circovirus type 2 (PCV2) vaccination of conventional pigs prevents viremia against PCV2 isolates of different genotypes and geographic origins. Vaccine.

[CR14] Franzo G, Cortey M, Segales J, Hughes J, Drigo M (2016). Phylodynamic analysis of porcine circovirus type 2 reveals global waves of emerging genotypes and the circulation of recombinant forms. Mol Phylogenet Evol.

[CR15] Fu Y, Sun X, Zhu H, Jiang R, Luo X, Yin L (2018). An optimized fed-batch culture strategy integrated with a one-step fermentation improves l-lactic acid production by *Rhizopus oryzae*. World J Microbiol Biotechnol.

[CR16] Grau-Roma L, Crisci E, Sibila M, López-Soria S, Nofrarias M, Cortey M, Fraile L, Olvera A, Segalés J (2008). A proposal on porcine circovirus type 2 (PCV2) genotype definition and their relation with postweaning multisystemic wasting syndrome (PMWS) occurrence. Vet Microbiol.

[CR17] Gresham A, Giles N, Weaver J (2000). PMWS and porcine dermatitis nephropathy syndrome in Great Britain. Vet Rec.

[CR18] Kamstrup S, Barfoed AM, Frimann TH, Ladekjær-Mikkelsen A-S, Bøtner A (2004). Immunisation against PCV2 structural protein by DNA vaccination of mice. Vaccine.

[CR19] Khayat R, Brunn N, Speir JA, Hardham JM, Ankenbauer RG, Schneemann A, Johnson JE (2011). The 2.3-angstrom structure of porcine circovirus 2. J Virol.

[CR20] Kim J, Chung H-K, Chae C (2003). Association of porcine circovirus 2 with porcine respiratory disease complex. Vet J..

[CR21] Kixmöller M, Ritzmann M, Eddicks M, Saalmüller A, Elbers K, Fachinger V (2008). Reduction of PMWS-associated clinical signs and co-infections by vaccination against PCV2. Vaccine.

[CR22] LaBarre DD, Lowy RJ (2001). Improvements in methods for calculating virus titer estimates from TCID50 and plaque assays. J Virol Methods.

[CR23] Lekcharoensuk P, Morozov I, Paul PS, Thangthumniyom N, Wajjawalku W, Meng X (2004). Epitope mapping of the major capsid protein of type 2 porcine circovirus (PCV2) by using chimeric PCV1 and PCV2. J Virol.

[CR24] Madson DM, Ramamoorthy S, Kuster C, Pal N, Meng XJ, Halbur PG, Opriessnig T (2008). Characterization of shedding patterns of Porcine circovirus types 2a and 2b in experimentally inoculated mature boars. J Vet Diagn Investig.

[CR25] Madson DM, Ramamoorthy S, Kuster C, Pal N, Meng XJ, Halbur PG, Opriessnig T (2009). Infectivity of porcine circovirus type 2 DNA in semen from experimentally-infected boars. Vet Res.

[CR26] Mahé D, Blanchard P, Truong C, Arnauld C, Le Cann P, Cariolet R, Madec F, Albina E, Jestin A (2000). Differential recognition of ORF2 protein from type 1 and type 2 porcine circoviruses and identification of immunorelevant epitopes. J Gen Virol.

[CR27] Martinez-Solis M, Herrero S, Targovnik AM (2019). Engineering of the baculovirus expression system for optimized protein production. Appl Microbiol Biotechnol.

[CR28] Misinzo G, Delputte PL, Meerts P, Lefebvre DJ, Nauwynck HJ (2006). Porcine circovirus 2 uses heparan sulfate and chondroitin sulfate B glycosaminoglycans as receptors for its attachment to host cells. J Virol.

[CR29] Mlera L, Bloom ME (2019). Differential zika virus infection of testicular cell lines. Viruses.

[CR30] Nawagitgul P, Morozov I, Bolin SR, Harms PA, Sorden SD, Paul PS (2000). Open reading frame 2 of porcine circovirus type 2 encodes a major capsid protein. J Gen Virol.

[CR31] Opriessnig T, Xiao CT, Halbur PG, Gerber PF, Matzinger SR, Meng XJ (2017). A commercial porcine circovirus (PCV) type 2a-based vaccine reduces PCV2d viremia and shedding and prevents PCV2d transmission to naive pigs under experimental conditions. Vaccine.

[CR32] Plackett RL, Burman JP (1946). The design of optimum multifactorial experiments. Biometrika.

[CR33] Pogranichniy R, Yoon K, Yaeger M, Vaughn E, Harmon K, Stammer R, Roof M (2004) Possible prevention of PMWS using inactivated PCV2 vaccine in CDCD pigs. In: Proceedings of the 18th IPVS congress, vol. 1, pp 55

[CR34] Segalés J, Allan GM, Domingo M (2005). Porcine circovirus diseases. Anim Health Res Rev..

[CR35] Stevenson GW, Kiupel M, Mittal SK, Choi J, Latimer KS, Kanitz CL (2001). Tissue distribution and genetic typing of porcine circoviruses in pigs with naturally occurring congenital tremors. J Vet Diagn Investig.

[CR36] Strober W (2001) Trypan blue exclusion test of cell viability. Curr Protoc Immunol. A. 3B. 1–A. 3B. 2 10.1002/0471142735.ima03bs21 18432654

[CR37] Thomas TL, Stolley PD, Stemhagen A, Fontham ETH, Bleecker ML, Stewart PA, Hoover RN (1987). Brain-tumor mortality risk among men with electrical and electronic jobs—a case–control study. J Natl Cancer Inst.

[CR38] Truong C, Mahe D, Blanchard P, Le Dimna M, Madec F, Jestin A, Albina E (2001). Identification of an immunorelevant ORF2 epitope from porcine circovirus type 2 as a serological marker for experimental and natural infection. Arch Virol.

[CR39] Weiss SA, Orr T, Smith GC, Kalter SS, Vaughn JL, Dougherty EM (1982). Quantitative measurement of oxygen consumption in insect cell culture infected with polyhedrosis virus. Biotechnol Bioeng.

[CR40] Wu PC, Chen TY, Chi JN, Chien MS, Huang C (2016). Efficient expression and purification of porcine circovirus type 2 virus-like particles in *Escherichia coli*. J Biotechnol.

[CR41] Young M, Cunningham G, Sanford E (2008) Performance of Ingelvac Circoflex^®^ vaccinated pigs in a subclinical PCVAD herd. In: Proceedings of the 20th congress IPVS, Durban, South Africa, vol 1, pp 25

