# Supplementary material for: Development of a simple and high-yielding fed-batch process for the production of porcine circovirus type 2 virus-like particle subunit vaccine
Source: AMB Express. 2019 Oct 11;9:164. doi: 10.1186/s13568-019-0880-8 (PMC6789058; doi:10.1186/s13568-019-0880-8)
Supplement: Supplementary file 1 — Additional file 1. Additional tables and figures. [file 13568_2019_880_MOESM1_ESM.docx]

Additional file

**Development of a simple and high-yielding fed-batch process for the production of** **porcine circovirus type 2 virus-like particle subunit vaccine**

Wenlong Cao ^a^†, Hui Cao^b^†, Xiaoping Yi ^a^* and Yingping Zhuang ^a^

^a^ school of biotechnology, East China University Of Science and Technology, Shanghai, 200237, China.

^b^ Zhejiang EBVAC Biotech CO., LTD., Hangzhou, Zhejiang, 310018, China.

† These authors contribute equally to this paper.

*Corresponding authors:

#Address correspondence to Xiaoping Yi, xpyi_ecust@sohu.com.

Additional tables:

**Table S1** Assigned concentrations of variables at different levels in Plackett–Burman design for cap protein production in Sf9 cells

| S.no | Variables with designate | Low level | High level |
| --- | --- | --- | --- |
|  |  | (-1) | (+1) |
| 1 | X1 Amino acids mixture^1^ | 0 | 3.88 g/L |
| 2 | X2 Hydrolysate |  |  |
|  | Yeast extract | 0 | 4 g/L |
|  | Rice extract | 0 | 2 g/L |
| 3 | X3 Lipids mixture^2^ | 0 | 1× |
| 4 | X4 Vitamin mixture^3^ | 0 | 2 g/L |
| 5 | X5 Inorganic Salts mixture^4^ | 0 |  |
| 6 | X6 disacchride |  |  |
|  | Maltose | 0 | 1 g/L |
|  | Sucrose | 0 | 1.65 g/L |
| 7 | X7 Organic acid |  |  |
|  | pyruvic acid | 0 | 6 mM |
|  | α- ketoglutaric acid | 0 | 6 mM |

^1^Amino acids mixture: 0.9 g/L Pro, 0.05 g/L His, 0.1 g/L Met, 0.08 g/L Lys, 0.05 g/L Ser, 0.6 g/L Thr, 1 g/L Trp, 1.2 g/L Cys, 0.9 g/L Tyr.

^2^Lipids mixture (Sigma-Aldrich, cat. L5146)

^3^Vanderzant vitamin mixture for insects (Sigma-Aldrich, cat.V1007)

^4^Inorganic Salts mixture: 0.4 mg/L (NH_4_)_6_MO_7_O_24_·4H_2_O, 0.5 mg/L CoCl_2_·6H_2_O, 2 mg/L CuCl_2_·2H_2_O, 5.5 mg/L FeSO_4_·7H_2_O, 0.2 mg/L MnCl_2_·4H_2_O, 0.4 mg/L ZnCl_2_

**Table S2** Plackett–Burman design for 7 variables with coded values and observed results of the maxium cell density in SFM

| S.No. | X1 | X2 | X3 | X4 | X5 | X6 | X7 | Maxium density |
| --- | --- | --- | --- | --- | --- | --- | --- | --- |
| 1 | -1 | 1 | 1 | -1 | 1 | -1 | -1 | 15.36 |
| 2 | 1 | 1 | -1 | 1 | -1 | -1 | -1 | 12.28 |
| 3 | 1 | 1 | 1 | -1 | 1 | 1 | -1 | 14.28 |
| 4 | -1 | 1 | -1 | -1 | -1 | 1 | 1 | 10.26 |
| 5 | 1 | 1 | -1 | 1 | 1 | -1 | 1 | 12.69 |
| 6 | 1 | -1 | 1 | -1 | -1 | -1 | 1 | 7.36 |
| 7 | -1 | -1 | -1 | -1 | -1 | -1 | -1 | 11.84 |
| 8 | -1 | -1 | 1 | 1 | 1 | -1 | 1 | 9.32 |
| 9 | 1 | -1 | 1 | 1 | -1 | 1 | -1 | 10.38 |
| 10 | 1 | -1 | -1 | -1 | 1 | 1 | 1 | 8.05 |
| 11 | -1 | -1 | -1 | 1 | 1 | 1 | -1 | 10.00 |
| 12 | -1 | 1 | 1 | 1 | -1 | 1 | 1 | 6.66 |

^a^The maxium cell density is the average value of the triplicate cultures with the unit of 106 cells/mL viable cells

**Table S3** Analysis of the results for Plackett-Burman design

| S.NO. | Variables | Coefficient | P value | Confidence |
| --- | --- | --- | --- | --- |
|  |  |  |  | Level (%) |
| X1 | Amino acids | -30.7 | 0.613 | 38.7 |
| X2 | Hydrolysate | 300.3 | 0.012 | 98.8 |
| X3 | Lipid | 28.0 | 0.642 | 35.8 |
| X4 | Vitamins | -39.7 | 0.519 | 48.1 |
| X5 | Inorganic salts | 24.7 | 0.106 | 59.4 |
| X6 | disaccharide | -96.3 | 0.175 | 92.5 |
| X7 | Organic acids | -172.7 | 0.015 | 98.5 |

Additional Figures:


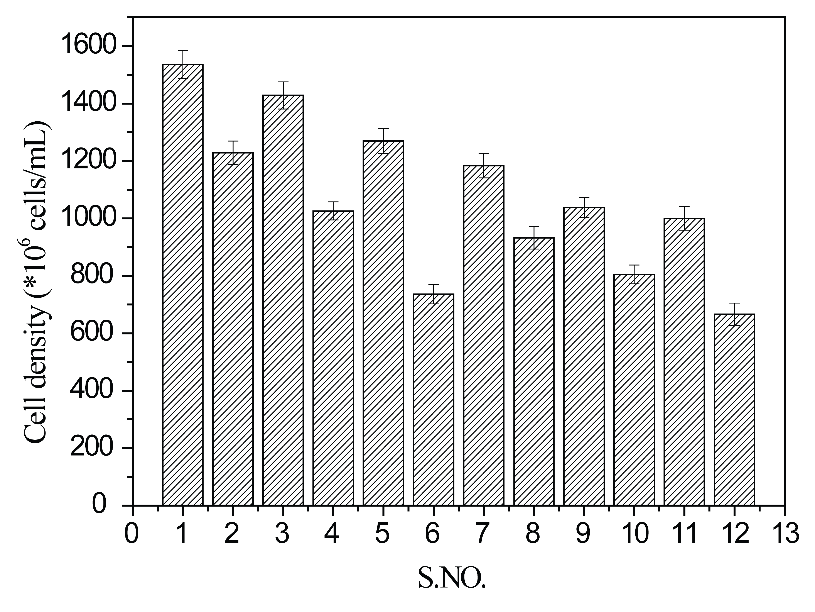


**Fig. S1** The maximum cell density in Sf9 cultures following nutrient concentrate additions were made according the Plackett-Burman design in the exponential growth phase. The data shown represents the average of three parallel flask experiments. Error bars represent the standard deviation.


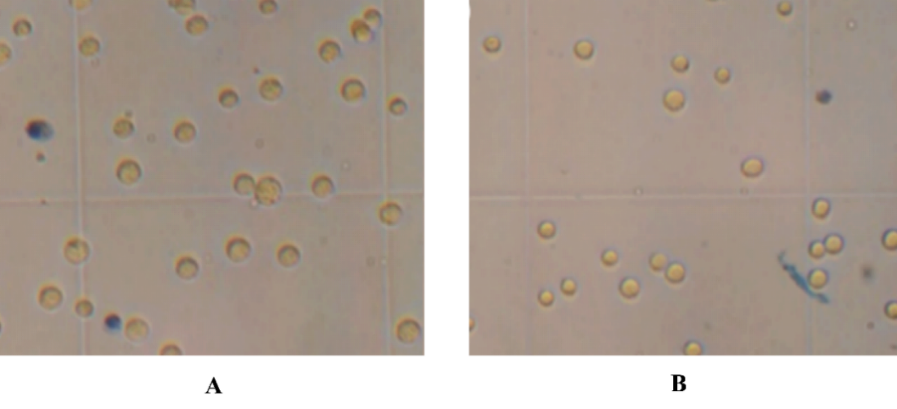


**Fig. S2** Image of Sf9 cells under a microscope (400×) in group C(A) and group D(B).
